# Anæsthetics in Short Operations on the Throat and Nose

**Published:** 1902-09-20

**Authors:** 


### ANÆSTHETICS IN SHORT OPERATIONS ON THE THROAT AND NOSE.

By short operations are meant those which can be performed during the time which elapses between the removal of the mask and the patient's return to consciousness. The most common of these are tonsillotomy and the removal of adenoids; posterior and anterior turbinectomy; removal of nasal spurs and polypi; and draining the antrum of Highmore through the alveoli. Now, as is pointed out by Chaldecott,[1] the enormous number of these operations performed renders the choice of an anæsthetic a matter of great importance, especially in view of the heavy mortality which occurs in this class of case from the use of chloroform. The operation of tonsillotomy and the removal of adenoids ought to be practically devoid of risk to life; yet he has collected a list of more than fifty recorded deaths under chloroform given for this operation, including adults and children, "cases in which the chloroform was given by specialists in anæsthetics, and cases in which it was administered by general practitioners and house surgeons." Here, he says, we have an operation which ought to be, and is in itself absolutely free from immediate danger, is perverted by the use of chloroform into one of the most immediately deadly of any in the long list of surgical operations, and he urges that the only way to escape this risk is to avoid the use of chloroform or any of its combinations in operations of this nature. Infants aged less than 12 months should be given ether on an open mask. Children from one to four years of age do not take nitrous oxide well, especially if they have large tonsils, but as a rule they take ether without any trouble at all from a Clover's inhaler, with or without the bag according to the ease with which they go under. For children from four to twelve years of age nitrous oxide will generally do, but if, when pressed, it should cause much jactitation and rigidity, it is better to give a little ether. In adolescents and adults the operation can be done under gas alone in most cases; indeed, a rapid operator will frequently remove in the time allowed by nitrous-oxide anæsthesia, tonsils, adenoids, and the posterior ends of the inferior turbinates; and what a rapid operator will do under gas alone an average operator will do easily under gas and ether.

[1] Lancet, Sept. 13.

